# Molecular Interactions between NR4A Orphan Nuclear Receptors and NF-κB Are Required for Appropriate Inflammatory Responses and Immune Cell Homeostasis

**DOI:** 10.3390/biom5031302

**Published:** 2015-06-29

**Authors:** Evelyn P. Murphy, Daniel Crean

**Affiliations:** UCD Veterinary Sciences Centre, Conway Insitute for Biomolecular and Biomedical Research, University College Dublin, Dublin 4, Ireland; E-Mail: evelyn.murphy@ucd.ie

**Keywords:** nuclear receptors, NR4A subfamily, NF-κB, inflammation, immune homeostasis

## Abstract

Appropriate innate and adaptive immune responses are essential for protection and resolution against chemical, physical or biological insults. Immune cell polarization is fundamental in orchestrating distinct phases of inflammation, specifically acute phase responses followed by resolution and tissue repair. Dysregulation of immune cell and inflammatory responses is a hallmark of multiple diseases encompassing atherosclerosis, rheumatoid arthritis, psoriasis and metabolic syndromes. A master transcriptional mediator of diverse inflammatory signaling and immune cell function is NF-κB, and altered control of this key regulator can lead to an effective switch from acute to chronic inflammatory responses. Members of the nuclear receptor (NR) superfamily of ligand-dependent transcription factors crosstalk with NF-κB to regulate immune cell function(s). Within the NR superfamily the NR4A1-3 orphan receptors have emerged as important regulators of immune cell polarization and NF-κB signaling. NR4A receptors modulate NF-κB activity in a dynamic fashion, either repressing or enhancing target gene expression leading to altered inflammatory outcome. Here we will discuss the pivotal role NR4A’s receptors play in orchestrating immune cell homeostasis through molecular crosstalk with NF-κB. Specifically, we will examine such NR4A/NF-κB interactions within the context of distinct cell phenotypes, including monocyte, macrophage, T cells, endothelial, and mesenchymal cells, which play a role in inflammation-associated disease. Finally, we review the therapeutic potential of altering NR4A/NF-κB interactions to limit hyper-inflammatory responses *in vivo*.

## 1. Introduction: Appropriate Inflammation and NF-κB Activity

Appropriate inflammatory responses to foreign pathogens or tissue injury are orchestrated by multiple cells types, including both resident (mast, endothelial, macrophage) and infiltrating (neutrophils, monocytes, macrophages, fibrocytes and T-cells) immune cells [1,2]. Innate immune sensors present on resident and infiltrating cells act to detect, initiate and propagate an inflammatory response [3,4]. These sensors are collectively known as pathogen recognition receptors (PRRs) and are activated by infectious agents such as foreign DNA, RNA, carbohydrates and non-infectious agents as occurs in “sterile” inflammatory processes [4,5]. Classes of PRRs include membrane bound sensors such as Toll-like receptors (TLRs), C-type lectins and cytoplasmic proteins such as NOD-like receptors (NLRs) and RIG-1-like receptors (RLRs) [3]. The major signaling pathways underlying PRR responses include mitogen activated protein kinases (MAPKs), IFN regulatory factors (IRFs) and NF-κB [3,6].

Subsequent to PRR activation resident cells secrete a range of mediators including histamine, bradykinin, prostaglandin (PGs), leukotrienes (LTs), cytokines and chemokines which co-operate to alter vascular permeability and promote immune cell recruitment and extravasation [7,8]. Polymorphonuclear (PMN) cell extravasation, driven primarily by leukotriene (LTB4), is the first line of defence of the innate immune system leading to phagocytosis and destruction of harmful materials [9]. Monocytes and monocyte-derived macrophages represent another critical immune cell, involved in both direct functional responses, such as phagocytosis, and eliciting dynamic downstream effects by secreting cytokines and chemokines which are important for regulating multiple stages of an appropriate inflammatory response [10]. A phrase coined “lipid class switching” takes place at this juncture, whereby lipid mediators responsible for the resolution of inflammation are secreted by cells within the inflammatory exudate [7]. These lipid mediators include lipoxins (LXs), resolvins (E and D series) (Rvs), protectins (Prot) and maresins (Mar) [7]. Collectively these resolving mediators inhibit PMN infiltration and extravasation, attenuate pro-inflammatory cytokine production, normalize chemokine gradients and promote PMN-CCR5 expression and subsequent non-phlogistic macrophage phagocytosis of apoptotic/CCR5 expressing PMNs [7,11]. Meanwhile tissue macrophages, regulatory T cells (Tregs), myofibroblast and fibrocyte cells promote tissue repair and remodelling as a result of growth factors such as TGF-β and ultimately tissue homeostasis [11,12].

Central to the control of all phases of the inflammatory response is the transcription factor NF-κB, and aberrant regulation of NF-kB signaling has been associated with pathologies characterised by altered immune responses and chronic inflammation (reviewed in [6]). Importantly NF-κB activity can also direct several anti-inflammatory processes such as appropriate leukocyte apoptosis and TGF-β release during the resolution phases of inflammation [13,14]. More than 25 years since the discovery of NF-κB, extensive studies provide a multifaceted understanding of the cell- and tissue-specific activation and downstream responses controlled by NF-κB [6]. However, as recently reviewed by *Hayden and Gosh*, and primarily due to the complexities of NF-κB activation and function, questions regarding molecular interactions at distinct levels of the NF-κB signaling pathway remain to be explored [6]. Over the past decade several members of the nuclear receptor superfamily (NR) have been uncovered as significant ligand-inducible proteins capable of influencing NF-κB activity in several cell types, including monocyte/macrophage cells. In this review article we discuss the role nuclear receptors play in mediating NF-κB regulation with a primary focus on the molecular interactions and crosstalk with the orphan nuclear receptor NR4A subfamily.

## 2. Nuclear Receptor (NR) Modulation of NF-κB Signaling

**Figure 1:**
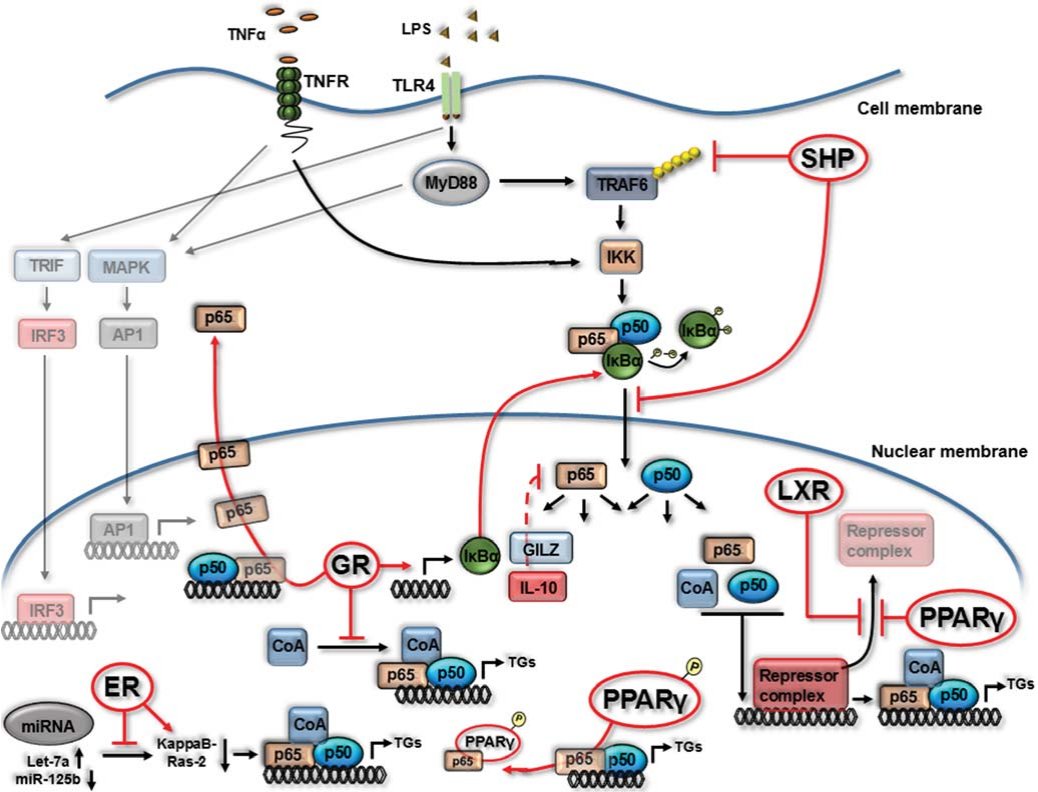
Nuclear receptors can alter NF-κB signaling and activity. Summary diagram depicting mechanisms by which nuclear receptors (NRs) impact the NF-κB signaling pathway downstream of TNF Receptor (TNFR) or Toll Like Receptor 4 (TLR4) as outlined within Section 2. Estrogen Receptor (ER), Glucocorticoid Receptor (GR), Peroxisome Proliferator-Activated Receptor gamma (PPARγ), Liver X Receptor (LXR), small heterodimer partner (SHP), transcriptional co-activators (CoA), phosphorylation (P), target genes (TGs). Red arrows display NR interactions with NF-κB (both inhibitory indicated by a T bar and stimulatory indicated by an arrow head **→**). Dashed arrow indicates multiple process involved.

NF-κB is a master regulator downstream of TLR and TNFα signaling pathways [15,16], and hence the molecular mechanisms by which NR’s impact on this pathway, and discussed herein, are primarily undertaken using TLR4 and TNFα-receptor (TNFR) stimulation (Figure 1). While in an inactivated state, NF-κB (p65/p50 heterodimer as depicted in Figure 1) is located in the cytoplasm bound with the inhibitory protein IκBα. Several extracellular signals including TNFα and LPS bind to integral membrane receptors (TNFR and TLR4) leading to activation of IκB kinase (IKK) which, in turn, phosphorylates the IκBα protein leading to ubiquitination and dissociation from the NF-κB complex. The activated NF-κB proteins translocate to the nucleus, recruit co-activators and subsequent binding to promoter regions to regulate target gene expression (Figure 1). Modulation of the NF-κB pathway by members of the NR superfamily occurs both upstream, within cytoplasmic signaling components, and in the nucleus during NF-κB DNA binding/transcriptional events (Figure 1). Given the central role of NRs in controlling NF-κB activity, the depletion or deletion of individual NRs can render myeloid cells to a hyper-NF-κB activated/pro-inflammatory state (Figure 1).

Glucocorticoids are potent anti-inflammatory agents and owe the majority of their anti-inflammatory functions to NF-κB inhibition [17]. The inhibitory effects of ligand activated glucocorticoid receptors (GR) on the NF-κB pathway occurs through the promotion of IκBα protein expression and subsequent NF-κB/p65 sequesterisation to the cytoplasm during TNFα exposure [18], a molecular mechanism also observed during TLR stimulation [19]. GR activation has also been shown to induce several anti-inflammatory factors known to inhibit NF-κB function in primary human PBMCs and immortalized macrophage cells, including IL-10 and glucocorticoid-induced leucine zipper (GILZ) [18,19,20,21,22]. Interestingly GILZ, induced by GR and IL-10 in macrophage cells, associates with NF-κB/p65 and prevents transcription from NF-κB-dependent regulatory elements [22]. Other mechanisms of GR inhibition of NF-κB activity include more rapid removal of NF-κB/p65 from the nucleus and inhibition of co-activating binding partners such as IRF3 [18,23].

Liver X receptor (LXR), a nuclear receptor involved chiefly in cholesterol homeostasis, has also been shown to impact on the NF-κB pathway [24,25,26,27]. Castrillo *et al.* demonstrate LPS-induced matrix metalloproteinase-9 (MMP-9) is inhibited by LXR in macrophage cells [27]. Interestingly, the study identifies a κB site on the MMP-9 promoter is essential for LXR inhibition, however, LXR does not alter IκBα expression or NF-κB/p65 DNA binding capacity. LXR activation in LPS stimulated macrophage cells inhibits the clearance of the NCoR/SMRT corepressor complex, thereby inhibiting NF-κB transcriptional activity [25]. This NCoR/SMRT co-repressor interaction was also identified as a primary mechanism for peroxisome proliferator-activated receptor gamma (PPARγ) inhibition of NF-κB activity (Figure 1) [25]. In dendritic cells (DCs) LXR appears to have the opposite effect on NF-κB activity and, during LPS exposure, positively increases activity leading to subsequent changes in target gene expression [28]. The mechanism in LPS-treated DCs was shown to be, in part, due to sustained IκBα phosphorylation by LXR and subsequent increase in NF-κB activation. Such differential responses highlight that specific cell types influence the molecular control of NF-κB activity by members of the NR family.

Thus the mechanisms by which NRs inhibit NF-κB activity are diverse, and on-going and future studies will uncover even greater levels of molecular complexity. Recently, using murine macrophages, the orphan nuclear receptor small heterodimer partner (SHP) has been shown to inhibit NF-κB activity at multiple stages of its pathway [29]. SHP can physically interact with NF-κB/p65, inhibiting its nuclear translocation, and can further modulate cytoplasmic TRAF6 ubiquitination/degradation, an important event as TRAF6 activates IKK in response to TLR4 activation (Figure 1). Furthermore, activation of estrogen receptor (ER) inhibits NF-κB activity in primary macrophages through alterations in miRNA-125b and Let-7a leading to the induction of kappaB-Ras2, an inhibitor of NF-κB signaling [30].

## 3. NR4A Nuclear Receptors and NF-κB Signaling

Emerging as significant regulators of inflammatory processes and immune homeostasis are the orphan nuclear receptor family 4A (NR4A). This subfamily is comprised of three members, NR4A1 (Nur77), NR4A2 (Nurr1) and NR4A3 (Nor-1) [2,31,32]. All three NR4A receptors are immediate early genes and expressed at high levels in a number of chronically inflamed human tissues and relevant mouse models, including atherosclerosis, arthritis, colitis and psoriasis [31,32,33]. To date no endogenous ligands have been described in the regulation of NR4A receptor activity, however pharmacological modulation of NR4A activity can be achieved with agents such as the anti-neoplastic agent, 6-mercaptopurine and a series of 1,1-bis(3-indolyl)-1-(*p*-substituted phenyl) methanes (C-DIMs) [34,35,36]. The NR4A1-3 receptors are constitutively active and their activity is controlled through transcriptional regulation, post translational modifications, protein interactions and subcellular localisation [31,32]. NF-κB is a pivotal regulator of NR4A1,2,3 gene expression in myeloid-derived cell types and multiple studies reveal a regulatory feedback role for NR4A’s in controlling NF-κB activity and pro-inflammatory target gene expression [37,38,39,40,41]. This regulation is primarily through repression of NF-κB transcriptional activity, as discussed herein.

NR4A1 deficiency highlights how crucial this receptor is for the development of Ly6C^low^ monocytes, particularly as Ly6C^low^ monocytes are involved in inflammatory resolution [39]. The impact of loss of Ly6C^low^ monocytes in NR4A1^−/−^ mice leads to enhancement of atherosclerosis in two distinct models of disease (Ldlr^−/−^ and ApoE^−/−^) [40]. The study further reveals that NR4A1 functions to regulate macrophage phenotypes *in vivo*, with concomitant increases in TLR expression and enhanced NF-κB-mediated target gene expression during TRL4 stimulation. Increased expression of IL-12, iNOS and enhanced responses to TLR agonists indicate polarisation of macrophages to an M1 pro-inflammatory phenotype. Using the selective and irreversible NF-κB inhibitor, BAY 11-7082, genes altered in NR4A1-deficient macrophages were confirmed to be NF-κB regulated targets. Furthermore, increased phosphorylation of NF-κB/p65 was measured in these cells affirming that NR4A1 deficiency can lead to increased NF-κB-dependent inflammatory activation in macrophage cells [40]. Recently NR4A1-dependent Ly6C^low^ monocytes have demonstrated a pivotal role in mediating intravascular homeostasis, in part by regulating necrosis of endothelial cells by neutrophils and subsequent phagocytic clearance [42].

Bone marrow specific deficiency of NR4A1 further reveals that bone marrow-derived macrophage (BMM) express higher levels of IL-12, IFNγ and SDF-1α with enhanced thioglycollate-elicited migration of macrophage and B cells. Again supporting a protective role for NR4A1 in hematopoietic cell in atherogenesis, transplantation of NR4A1^−/−^ BMM to low density lipoprotein receptor-deficient (Ldlr^−/−^) mice enhances atherosclerosis with increased lesion size and enhanced macrophage and T cell numbers [41]. These observations support earlier studies by the *de Vries* group demonstrating how ectopic expression of NR4A1, 2 or 3 in human macrophage cells attenuates pro-inflammatory cytokine (IL-6, IL-1β and IL-8) and chemokine (MIP1α and MCP-1) synthesis [38]. Additionally in similar human cells, knockdown of NR4A1 or NR4A3 expression results in enhanced TLR4-stimulated production of IL-1β, IL-8 and MCP-1 [38]. Macrophage cell phenotype is not only characterised by cytokine/chemokine expression profile but rather a combination of secreted mediators and specific surface marker expression patterns. Interestingly NR4A1^−/−^ macrophages isolated from atherosclerotic murine models display enhanced pro-inflammatory surface marker expression (MHC class II) and reduced anti-inflammatory marker expression (Arginase-1) [40].

**Figure 2:**
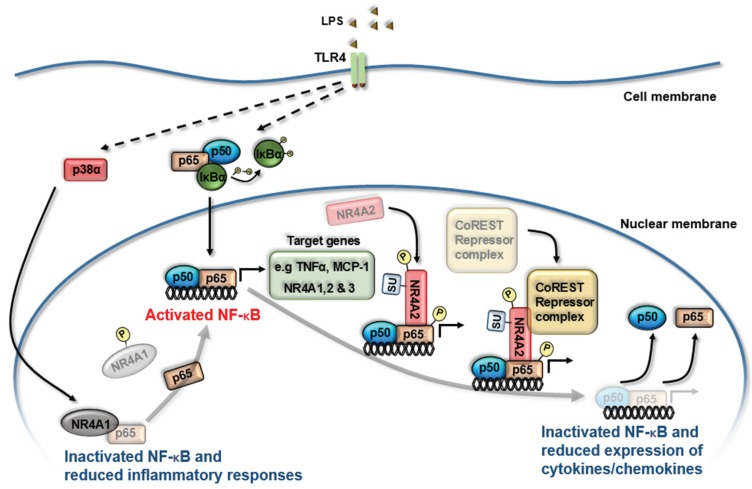
NR4A receptors regulate NF-κB activity to limit inflammatory responses. Summary diagram displaying the molecular mechanisms by which NR4A receptors regulate NF-κB transcriptional activity downstream of Toll Like Receptor 4 (TLR4) activation. LPS treatment of myeloid-derived cells triggers the transcriptional induction of all three NR4A receptors via NF-κB (p50/p65 heterodimer) binding to κB sites within their promoter regions. Enhanced NR4A1 and NR4A2 expression functions to limit the inflammatory response by altering NF-κB-dependent regulation of cytokine and chemokine gene expression. During inflammation NR4A2 becomes SUMOylated (by protein inhibitor of activated STAT4) and phosphorylated (by nemo-like kinase) which in turn binds to phosphorylated NF-κB/p65 (by glycogen synthase kinase-3) on target gene promoter regions, leading to the recruitment of a repressor complex known as Co-REST. LPS/TLR-activated p38α phosphorylates NR4A1 to reduce NR4A1-dependent suppression of a hyper-inflammatory response through NR4A1 inhibition of NF-κB/p65 binding to κB sites within promoter regions of target genes. SUMOylation (SU), phosphorylation (P).

The molecular mechanism by which NR4A receptors impact on NF-κB activity came from the initial study of NR4A2 activity in microglia cells under TLR4 stimulation [43]. Depleted NR4A2 expression results in heightened pro-inflammatory responses in microglial cells. Within this cell model and following TLR4 stimulation, NR4A2 becomes SUMOylated (by protein inhibitor of activated STAT4) and phosphorylated (by nemo-like kinase) which in turn binds to phosphorylated NF-κB/p65 (by glycogen synthase kinase 3) on target gene promoter regions, leading to the recruitment of a repressor complex known as Co-REST [43]. Association of this repressor complex promotes clearance of NF-κB/p65 from the promoter of targets genes such as TNFα and subsequent dampening of pro-inflammatory responses [43] (Figure 2). NR4A2 recruitment to the promoter regions of pro-inflammatory gene targets, TNFα and iNOS, was further confirmed using an *in vivo* model. Cell-specific knockdown of NR4A2 renders the cells to a hyper-inflammatory state with cellular responses further heightened following TLR4 stimulation, displaying augmented TNFα, iNOS and IL1β production [43]. Altered expression of NR4A2 and -3 has been shown to guide macrophage polarization [44,45,46] with adoptive transfer experiments revealing that NR4A2 confers increased survival in endotoxin (LPS) induced sepsis [44]. Similar molecular mechanisms by NR4A1 are employed in controlling immune homeostasis in LPS-induced sepsis [47]. NR4A1^−/−^ mice display an increased sensitivity to LPS-induced sepsis and death. In peritoneal macrophage cells NR4A1 functions to limit NF-κB activity through the inhibition of NF-κB/p65 binding to κB binding sites on target gene promoters. Interestingly, the phosphorylation of NR4A1 by LPS-activated p38α leads to loss of NR4A functional activity, suggesting that targeting the interaction of p38α and NR4A1 would permit NR4A1 mediated control of hyper-inflammatory responses (Figure 2) [47].

Collectively these studies confirm that NR4A1-3 receptors are intimately involved in monocyte cell differentiation, with the loss of receptor activity promoting a hyper-inflammatory cell response. This NR4A-dependent pro-inflammatory activation state is mainly attributed to the loss of appropriate control of NF-κB signaling and NF-κB/p65 functional activity [39,40,43]. Importantly NR4A1 has also been shown to promote NF-κB activity through the activation of IKKi in murine macrophage cells [48]. Additionally, within models of vascular injury, NR4A3/Nor-1 can protect or potentiate neointima formation following insult leading to diverse outcomes [49,50,51,52,53]. Such differences may underlie additional complexities to the function(s) of NR4A receptors, as a consequence of tissue environment, cell or stimuli specific effects. Hence to clarify the role of NR4A3 in mediating the response of vascular smooth muscle cells to inflammatory stimuli, *in vivo* cell specific gain and loss of function studies have recently been undertaken [50,51]. NR4A3 was shown to direct and limit the inflammatory response by inhibiting NF-kB/p65 activation, nuclear translocation and target gene expression [51].

## 4. NR4A Control of Acute Inflammation, Inflammatory Resolution and Tissue Repair

Target genes and physiological functions of NR4A receptors are cell type and context dependent. Therefore, and as summarised below, the study of distinct cell types in models of inflammation-associated disease is proving critical to uncovering NR4A receptor involvement in tissue responses that promote inflammatory resolution including acute inflammation, vascular permeability, angiogenesis and tissue remodelling.

### 4.1. NR4A1 is Crucial for the Development of Ly6C^low^ Reparative Monocytes and Has an Anti-Inflammatory Effect in Monocyte/Macrophage Cells

Treatment of monocyte/macrophage cells with LPS, cytokines, or oxidized lipids triggers the transcriptional induction of all three NR4A receptors [37,44]. Functional studies demonstrate both pro- and anti-inflammatory functions for NR4A receptors suggesting that the temporal expression levels and activity of these receptors in monocyte/macrophage cells may permit differential effects on gene expression and transcriptional outcomes. In models of acute myocardial infarction (MI), healing and tissue remodeling requires monocyte/macrophage cells which persist for days in the infarct zone and contribute to diverse functions including inflammation, proteolysis, phagocytosis, angiogenesis and collagen deposition [54,55]. It is proposed that heterogeneity of monocyte subtypes leads to biphasic cellular and molecular events that control these functions and influence healing and remodeling [56]. Recently a role for NR4A1 has been demonstrated within a murine model of acute MI. Mice lacking NR4A1 on hematopoietic cells, display increased inflammatory responses and compromised healing responses following acute MI. Enhanced accumulation of NR4A1^−/−^ monocytes and macrophage cells are measured in myocardial infarct tissue. In the absence of NR4A1, Ly-6C^high^ monocytes express high levels of CCR2, infiltrate myocardium in excessive numbers and differentiate to abnormal inflammatory macrophages [56]. These studies indicate that NR4A1 activity is required to balance an early monocyte-dominant inflammatory response and a later macrophage-dominant reparative phase. Such observations enrich our mechanistic understanding of the diverse molecular events controlled by NR4A receptors within monocyte/macrophages which may have biological relevance beyond MI. Regarding the functional role of NF-κB activity in MI, studies demonstrate NF-κB activation during acute injury is protective whereas prolonged activation leads to tissue damage [57]. It is becoming more evident that the local environment influences the differentiation stage/function of immune cells, thus determining the cell specific contribution NR4A receptors play in guiding inflammatory responses and outcomes [56,57].

### 4.2. Generation and Maintenance of Regulatory T Cells by NR4A Receptors

In CD4/CD8 double-positive thymocytes and T cell hybridomas, NR4A expression is induced through the T cell receptor (TCR) and is implicated in activation-induced apoptosis during negative selection [58]. Overexpression of NR4A1 in the human T cell leukemia cell line (Jurkat) can repress expression of IL-2 via negative regulation of NF-κB activity [59]. Mice lacking all three NR4A family members fail to produce regulatory T (Treg) cells and die prematurely due to uncontrolled systemic multi-organ inflammation resembling the phenotype of Forkhead transcription factor, *Foxp3*, deficient mice [60]. Ectopic expression of NR4A2 induces the expression of *Foxp3,* which through the suppression of effector cytokine production, plays a critical role in the differentiation, maintenance and effector functions of Treg cells [61]. Alternatively, chromatin immunoprecipitation of endogenous NR4A proteins reveals that each NR4A family member can bind the *Foxp3* promoter in stimulated naive T cells. Thus, all three NR4A transcription factors are potentially able to activate Foxp3 expression to promote Treg development and functions [60]. Recent work by the *Hedrick* group has further established a key role for NR4A1 in regulating appropriate CD8+ T cell development through modulation of *Runx3* [62]. The authors reveal during CD8+ T cell development NR4A1 recruits the co-repressor CoREST complex, binds to a distal promoter region of the *Runx3* gene, and subsequent gene repression [62].

As highlighted in Figure 1, TRAF6 is a key upstream regulator of NF-κB signaling. TRAF6 has been shown to play an essential role in activating NF-κB in T cells, and recent studies indicate that this protein also plays a critical role in the development of Treg cells. TRAF6 deficiency leads to reduced expression and stability of Foxp3 and a rapid conversion of Foxp3^+^ Tregs to Foxp3^−^ cells [63]. Molecular interactions between NR4A/TRAF6/NF-κB in Foxp3 expression during Treg development and maintenance is unknown and remains to be studied.

### 4.3. Role of NR4A Receptors in Maintaining Endothelial Cell Homeostasis

Changes in endothelial cell (EC) activation, vascular permeability and angiogenesis are early histopathological events that facilitate leukocyte ingress into inflamed tissue. NR4A1 protects ECs from TNFα- and IL1β-induced endothelial activation. Attenuation is mediated through the induction of IκBα and subsequent inhibition of NF-κB activity and transcriptional regulation of adhesion molecule expression [64]. NR4A1 transcriptional activity has also been shown to regulate vascular permeability *in vitro* and *in vivo* by enhancing endothelial NO synthase and down-regulating several EC junction proteins that are required to maintain vascular homeostasis [65,66].

The dynamic balance of positive and negative effectors of angiogenesis is of critical importance in the regulation of new vessel formation and stability in health and disease. NR4A1 transcriptional activity is both necessary and sufficient for VEGF-A-Induced proliferation and survival of cultured endothelial cells (ECs) and for angiogenesis *in vivo*, while depletion of NR4A2 expression attenuates VEGF-A-induced endothelial proliferation, migration and *in vivo* matrigel angiogenesis [67,68]. A role for NR4A receptors in regulating the expression of the endogenous angiogenesis inhibitor, thrombospondin-1 (TSP-1), has been recently explored. In human inflammatory arthritis the expression pattern of TSP-1 is significantly altered and these levels can be restored in patients responding to TNFα inhibition [69]. Further studies have uncovered an inverse relationship between NF-κB activity and TSP-1 expression *in vivo* and *in vitro* studies confirm NF-κB-dependent repression of TSP-1 promoter activity, mRNA and protein expression [69]. NR4A2 functions as a transcriptional repressor of TSP-1 in human synovial tissue leading to reduced TSP-1 expression [69]. The identification of TSP-1 as a target of NR4A2 activity complements and enhances studies proposing a pro-angiogenic role for the NR4A subfamily of receptors *in vivo*. Interestingly, another member of this nuclear receptor family, PPAR, has been shown to modulate TSP-1 during early inflammation. PPARα^−/−^ in leukocytes results in enhanced TSP-1 expression while PPARγ ligands greatly augment the pro-apoptotic effects of the TSP-1 derived peptide, ABT510 [70,71].

Physiologic angiogenesis plays an important role in tissue repair/wound healing and the involvement of endothelial NR4A1-3 receptor activity in skin wound healing is under review [72,73]. Initial studies indicate that NR4A1^−/−^ mice have comparable skin healing to wild-type littermates [73]. However the healing process is significantly delayed in in EC-NR4A1-dominant negative mice, in which a dominant negative NR4A1 mutant is inducible and specifically expressed in mouse ECs. Taken together these data suggest that NR4A1 and family members, NR4A2 and -3, play an effective and redundant role in normal skin would healing [73].

### 4.4. NR4A Receptors Mediate Growth Factor and Cytokine Responses in Mesenchymal Cells

The transdifferentiation of epithelial cells into motile mesenchymal cells (epithelial mesenchymal transition—EMT) is integral in development, stem cell behavior and wound healing, and contributes pathologically to fibrosis, inflammatory arthritis and cancer progression [74]. During EMT the reprogramming of gene expression are initiated and controlled by intracellular pathways that respond to extracellular mediators including pro-inflammatory cytokines and growth factors of which TGF-β has a predominant role [74].

Migration and invasion of mesenchymal derived fibroblast-like synoviocytes (FLSs) are critical in the pathogenesis of rheumatoid arthritis (RA). Enhanced NR4A2 activity induces a phenotypic shift in normal FLS that parallels the cellular transformation and hyperplasia observed during the progression of inflammatory arthritis [75]. Through the generation of stable and transient NR4A2 expressing FLS, an *in vitro* cell system that reflects the characteristics of activated primary FLS was created. NR4A2 over-expressing cell lines express high VEGF-A and IL-8 levels, with significantly reduced TSP-1 mRNA and secreted protein levels [69]. Further studies confirm that TNFα control of IL-8 expression and secretion by FLS is through a co-operative NR4A2/NF-κB driven process [69,76]. NF-κB inhibition attenuates TNFα-driven IL-6, IL-8 and MCP-1 in FLSs [77] and NF-κB inhibition is a protective therapeutic preventing the development of collagen induced arthritis (CIA) [77]. Such observations are supported by NR4A1 and NR4A2 gain of function experiments which reveal enhanced IL-6 and IL-8 production by bone marrow derived mesenchymal stromal cells [78]. In breast cancer NR4A1 activity is involved in mediating inflammation-induced epithelial to mesenchymal transition and breast metastasis [79]. Inflammatory mediators induce NR4A1 expression which drives TGF-B mediated breast cell migration, invasion and metastasis *in vitro* and *in vivo*. Notably, NR4A1 expression levels are elevated in breast cancer patients with high immune cell infiltration and levels correlate with poor prognosis [79]. Recent characterization of experimentally induced skin, lung, liver and kidney fibrosis in mice further identifies a regulatory role of NR4A1 in controlling aberrant TGF-β signaling [72]. Thus, disease stage, sustained chronic inflammatory stimuli, including the balance of pro-inflammatory cytokines and TGF-β, may determine NR4A levels of activity which directs their functional role(s) in disease progression.

## 5. Therapeutic Potential of Altering NR4A/NF-κB Interactions to Control Inflammation

The ability of NR4A1-3 receptors to integrate and limit inflammatory signaling and promote tissue repair makes these receptors potential targets for therapeutic intervention. Endogenous ligands have yet to be identified for members of the NR4A subfamily. These receptors may be termed as “true” orphan receptors due to the presence of large hydrophobic side chains within their ligand binding domains (LBD) that may preclude ligand binding [32,34,36,80]. Modulating NR4A receptor activity has focused on altering expression levels, modulating cellular localization and impacting on interactions with co-regulatory proteins [32,36]. Pharmacological modulation of NR4A activity can be achieved with the anti-neoplastic agent, 6-mercaptopurine, and related compounds including adenosine [35,46]. A structurally different class of NR4A ligands include a series of 1,1-bis(3-indolyl)-1-(*p*-substituted phenyl) methanes (C-DIMs) which have been identified as potential NR4A1/2 agonists [34]. Recent studies reveal that in microglial cells [81], C-DIMs can increase NR4A2 nuclear localisation, stabilization of Co-REST and NCoR2 complexes and subsequent inhibition of NF-κB/p65 signaling, supporting the earlier NR4A2/NF-κB study by *Glass* and colleagues [43]. The *in vivo* therapeutic potential of targeting NR4A/NF-κB interactions to control immune homeostasis has recently been uncovered (Figure 2) [47]. A chemical compound n-pentyl 2-[3,5-dihydroxy-2-(1-nonanoyl)-phenyl]acetate (PDNPA) is shown to target the LBD of NR4A1 and prevents phosphorylation of NR4A1 by TLR-activated p38α. Loss of NR4A1 phosphorylation restores and maintains NR4A1 suppression of a hyper-inflammatory response through NR4A1 inhibition of NF-κB/p65 binding to κB sites within promoter regions of target genes [47]. Thus, pharmacological modulation of NR4A receptors with therapeutic potential to control hyper-inflammatory responses is developing and extremely promising. However, as revealed through the study of distinct inflammatory-associated diseases, cell type, cell context and disease stage can influence NR4A receptor functions, and consequently effective therapeutic NR4A modulation to ensure appropriate inflammatory resolution and repair may be challenging.

## 6. Conclusions

NR4A1-3 orphan nuclear receptors have emerged as significant regulators of the inflammatory response modulating NF-κB signaling and activity in distinct cell types including monocyte/macrophage cells. NR4A receptors are required in inflammatory disease initiation and progression, where they function as early response regulators, controlling the extent of the inflammatory response and promoting inflammatory resolution. Given the pivotal role myeloid cells play in chronic inflammatory diseases NR4A receptors are an attractive target for therapeutic intervention. However, cognizance of the observations that cell-type and cellular microenvironment can alter NR4A receptor activity and influence their biological roles, the study of appropriate *in vivo* models of inflammatory disease will be important to ascertain their applicability as therapeutic targets.
